# Structural Insights into the Mechanism for Recognizing Substrate of the Cytochrome P450 Enzyme TxtE

**DOI:** 10.1371/journal.pone.0081526

**Published:** 2013-11-25

**Authors:** Feng Yu, Minjun Li, Chunyan Xu, Zhijun Wang, Huan Zhou, Min Yang, Yaxing Chen, Lin Tang, Jianhua He

**Affiliations:** 1 Shanghai Institute of Applied Physics, Chinese Academy of Sciences, Shanghai, China; 2 Shanghai Institutes for Biological Sciences, Chinese Academy of Sciences, Shanghai, China; University of Bologna & Italian Institute of Technology, Italy

## Abstract

Thaxtomins, a family of phytotoxins produced by *Streptomyces spp.*, can cause dramatic plant cell hypertrophy and seedling stunting. Thaxtomin A is the dominant form from *Streptomyces scabies* and has demonstrated herbicidal action. TxtE, a cytochrome P450 enzyme from *Streptomyces scabies 87.22*, catalyzes direct nitration of the indolyl moiety of L-tryptophan to L-4-nitrotryptophan using nitric oxide, dioxygen and NADPH. The crystal structure of TxtE was determined at 2.1 Å resolution and described in this work. A clearly defined substrate access channel is observed and can be classified as channel 2a, which is common in bacteria cytochrome P450 enzymes. A continuous hydrogen bond chain from the active site to the external solvent is observed. Compared with other cytochrome P450 enzymes, TxtE shows a unique proton transfer pathway which crosses the helix I distortion. Polar contacts of Arg59, Tyr89, Asn293, Thr296, and Glu394 with L-tryptophan are seen using molecular docking analysis, which are potentially important for substrate recognition and binding. After mutating Arg59, Asn293, Thr296 or Glu394 to leucine, the substrate binding ability of TxtE was lost or decreased significantly. Based on the docking and mutation results, a possible mechanism for substrate recognition and binding is proposed.

## Introduction

Cytochromes P450 superfamily (CYP) is a large and diverse group of enzymes containing a heme cofactor which participate in a variety of physiological roles including hormone and secondary metabolite biosynthesis, as well as xenobiotic metabolism[1,2]. Most of CYPs catalyze the insertion of an oxygen atom from dioxygen into the carbon-hydrogen bonds of organic compounds, while the other oxygen atom reacts with protons to form water. This reaction is referred to as the monooxygenase reaction. However, many other types of catalytical activity of CYPs have been demonstrated, including C-C bond cleavage, oxidative dealkylation, C-C bond formation, various ring alterations, and the reduction of nitric oxide to nitrous oxide[1,3,4].

**Figure 1:**
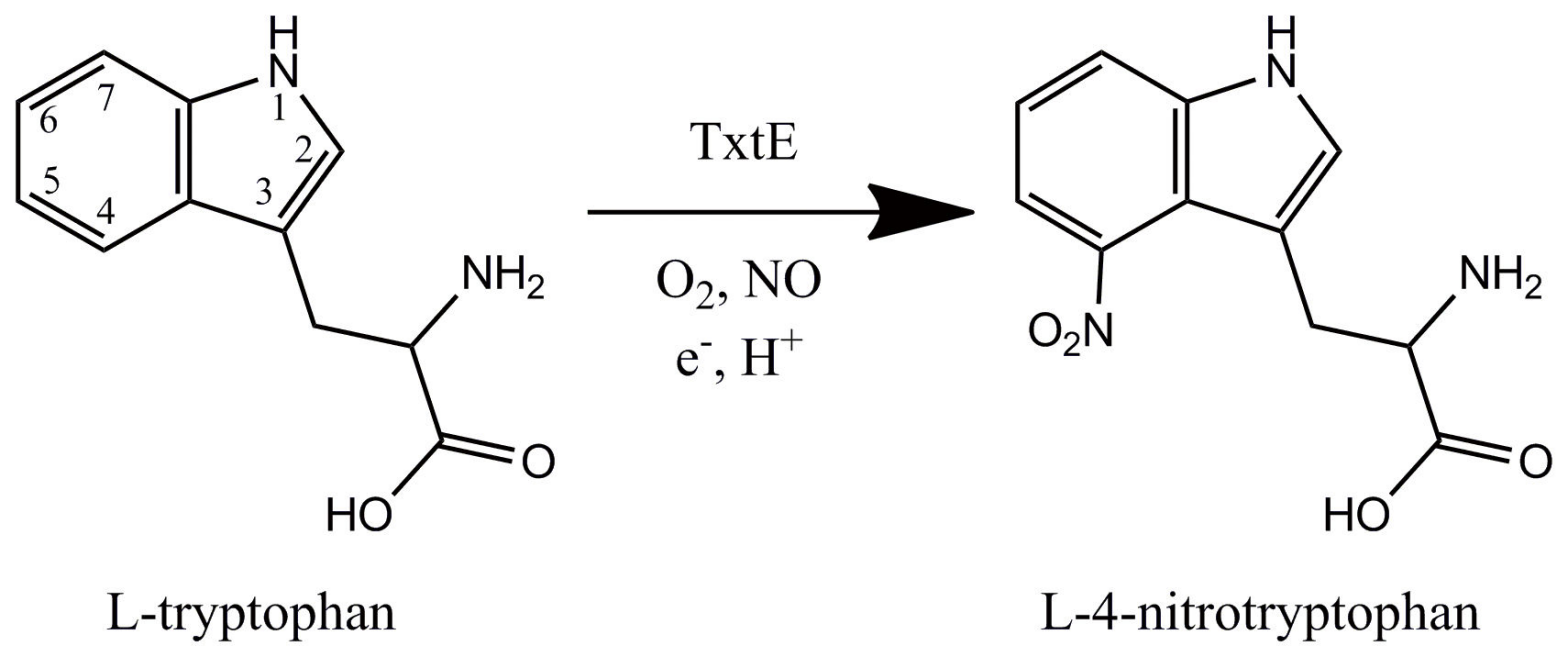
TxtE catalyzes direct nitration of the indolyl moiety of L-tryptophan to L-4-nitrotryptophan.

Thaxtomins, a family of phytotoxins produced by *Streptomyces spp.*, can cause dramatic plant cell hypertrophy and seedling stunting[5]. Thaxtomin A is the dominant form from *Streptomyces scabies* which also has demonstrated herbicidal action. Until recently, six proteins were identified in the Thaxtomin A biosynthesis pathway: TxtA and TxtB are non-ribosomal peptide synthases[6,7]; TxtC[8] and TxtE[9] are CYPs; TxtD is a nitric oxide synthase that produces nitric oxide from L-arginine[10]; TxtR is a cellobiose-responsive pathway specific activator[11]. TxtE catalyzes direct nitration of the indolyl moiety of L-tryptophan (L-Trp) to L-4-nitrotryptophan using nitric oxide, dioxygen and NADPH (Figure 1), which is the key step of Thaxtomin A biosynthesis. Although NO-related nitration is not an uncommon chemical process in organisms, particularly for tyrosine nitration[12], TxtE is the first reported enzyme that catalyzes a direct nitration reaction specifically in a biosynthetic pathway and thus it can potentially be developed for industrial applications. TxtE catalyzes regiospecific nitration of L-tryptophan. D-tryptophan is not the substrate of TxtE. Tryptophan nitration can take place at positions 1, 2, 4, 5, 6 and 7 of the indolyl moiety of tryptophan[13]. However, TxtE only catalyzes a nitration reaction at the 4-position of indolyl moiety. In the reaction products, one oxygen atom of the nitro group and the oxygen atom of the water molecule come from dioxygen. Combined with the catalytic mechanism of CYPs, it was proposed that TxtE may activate dioxygen and nitric oxide to form a peroxynitrite[9].

In this paper, we report the crystal structure of TxtE in substrate-free form. TxtE shows an open conformation. A clearly defined substrate access channel and a potential proton transfer pathway were observed. According to docking and mutagenesis experiments, we propose a possible substrate recognition and binding mechanism.

## Materials and Methods

### Protein expression and purification

The full-length TxtE DNA sequence from *S. scabies 87.22* (406 amino acids) was subcloned into plasmid pET-28a, which produced an N-terminal His-tagged protein with a thrombin digestion site. The R59L (TxtE-R59L), Y89F (TxtE-Y89F), N293L (TxtE-N293L), T296L (TxtE-T296L) and E394L (TxtE-E394L) mutants were produced by applying mutagenic PCR to the pET28a-TxtE vector according to QuikChange protocol.

Wild-type TxtE protein was expressed in *Escherichia coli* Rosetta(DE3) plysS cells in LB broth medium for 12 hours at 20°C with 1 mM IPTG induction. The resulting cell pellet was re-suspended in buffer A (20 mM Tris-HCl pH8.0, 300 mM NaCl, 5% Glycerol). Cells were lysed by passing through a microfluidizer (18,000 psi) twice, and then the lysate was centrifuged at 30,000 g for 30 minutes. The supernatant was loaded into a 5 mL Ni-IDA column (GE Healthcare) which had been equilibrated with buffer A, and then eluted by using buffer B (20 mM Tris-HCl pH 8.0, 300 mM NaCl, 300 mM imidazole, 5% glycerol). After dilution with buffer C (20 mM Tris-HCl pH 8.0, 5% glycerol), the sample was loaded into HiTrap Q FF and eluted by using a linear gradient. Finally, TxtE was loaded into a Superdex 75 16/60 column which was equilibrated with buffer D (25 mM Tris-HCl pH 8.0, 150 mM NaCl and 5% glycerol). The resulting protein peak was concentrated to 15 mg/mL by using a 10 kDa MWCO Amicon Ultra. Aliquots were snap frozen in liquid nitrogen and stored at −80°C until they were used for crystallization. TxtE-R59L, TxtE-Y89F, TxtE-N293L, TxtE-T296L and TxtE-E394L mutant proteins were expressed and purified using the same method as mentioned above.

### Crystallization and structure determination

Before crystallization, fresh DTT and imidazole pH 6.5 were added to protein solution. The final concentrations were 2 mM and 200 mM, respectively. Crystals of TxtE were grown by the hanging-drop vapor diffusion method at 18°C. Protein solution was mixed with equal volumes of reservoir solution containing 20% MPD and 20% PEG6000. Crystals were cryo-protected with cryoprotectant before freezing in liquid nitrogen.

X-ray diffraction data were collected at beamline BL17U1 of the Shanghai Synchrotron Radiation Facility. Data were processed using the HKL2000 software suite[14]. The TxtE structure was solved by molecular replacement using Phaser[15] with P450BIOI[16] (PDB: 3EJB) as the search model. After molecular replacement, maximum likelihood-based refinement of the atomic positions and temperature factors were performed with PHENIX[17], and the atomic model was fit with the program Coot[18]. The stereochemical quality of the final model was assessed with MolProbity[19]. Crystallographic statistics for the final model are shown in Table 1. The substrate access channel was analyzed using Caver[20]. Figures were prepared with PyMOL[21].

**Table 1:** Data collection and refinement statistics.

**Parameter**	**Values**
**Data collection**	
Space group	P1
*a* (Å)	46.3
*b* (Å )	52.6
*c* (Å)	88.2
α, β, γ (°)	77.8, 82.2, 68.4
Resolution (Å)	50.00 – 2.10 (2.14 – 2.10)**^*a*^**
*R*_merge_ (%)	4.6 (37.7)**^*a*^**
<*I*>/<σ*I*>	28.7 (5.5)**^*a*^**
Completeness (%)	96.7 (96.5)**^*a*^**
Redundancy	3.9 (3.9)**^*a*^**
**Refinement**	
No. reflections	40,644
*R*_work_/*R*_free_ (%)	19.64/23.43***^b^***,***^c^***
No. atoms	
Protein	6141
Ligand	101
Water	339
RMSD	
Bond lengths (Å)	0.005
Bond angles (°)	1.255
Ramachandran plot (%)	
Favored	97.70
Allowed	2.05
Outliers	0.26

^a^ Values in parentheses are for the highest resolution shell.

^b^ $R=\sum_{hkl}\left|F_{obs}-F_{calc}\right|/\sum_{hkl}F_{obs}$

^c^ *R*_free_, calculated the same as *R*_work_, but from a test set containing 5% of data excluded from the refinement calculation.

### Substrate docking calculations

L-tryptophan was docked into the structure of TxtE in the zwitterionic form using AutoDock 4.2[22]. Imidazole and water molecules were removed before docking. All side chains were set to rigid body and grid spacing was set to 1 Å. Other parameters were with their default values. The three lowest-energy docking solutions from 2,500,000 search results were chosen since they all showed relevant enzyme-substrate interactions.

### L-tryptophan binding assays of wild-type TxtE, TxtE-R59L, TxtE-T296L and TxtE-E394L

Purified wild-type TxtE, TxtE-R59L, TxtE-T296L and TxtE-E394L were exchanged to buffer E (25 mM Tris-HCl pH 8.0) by ultrafiltration. Finally, protein was diluted to about 15 μM using buffer E. Spectra were recorded between 325 nm and 500 nm with or without L-tryptophan using a UV-vis spectrophotometer (Chirascan, Applied PhotoPhysics, UK). The final concentration of L-tryptophan was 1 mM.

### Determination of dissociation constants of L-Tryptophan from wild-type TxtE, TxtE-Y89F and TxtE-N293L

Purified wild-type TxtE, TxtE-Y89F, and TxtE-N293L were exchanged to buffer E (25 mM Tris-HCl pH 8.0) by ultrafiltration. Finally, the protein was diluted to about 8 μM using buffer E. L-tryptophan was added to the sample cuvette until the protein was saturated. The added substrate solution did not exceed 2% of the total volume. The protein solution without L-tryptophan was served as control. This procedure was repeated three times for each sample. The average values of the difference in absorbance of each spectrum at 390 nm and 420 nm were plotted against the concentration of the substrate. The data were fitted to a one site binding model using Qtiplot.

## Results

### Overall structure and the substrate access channel

**Figure 2:**
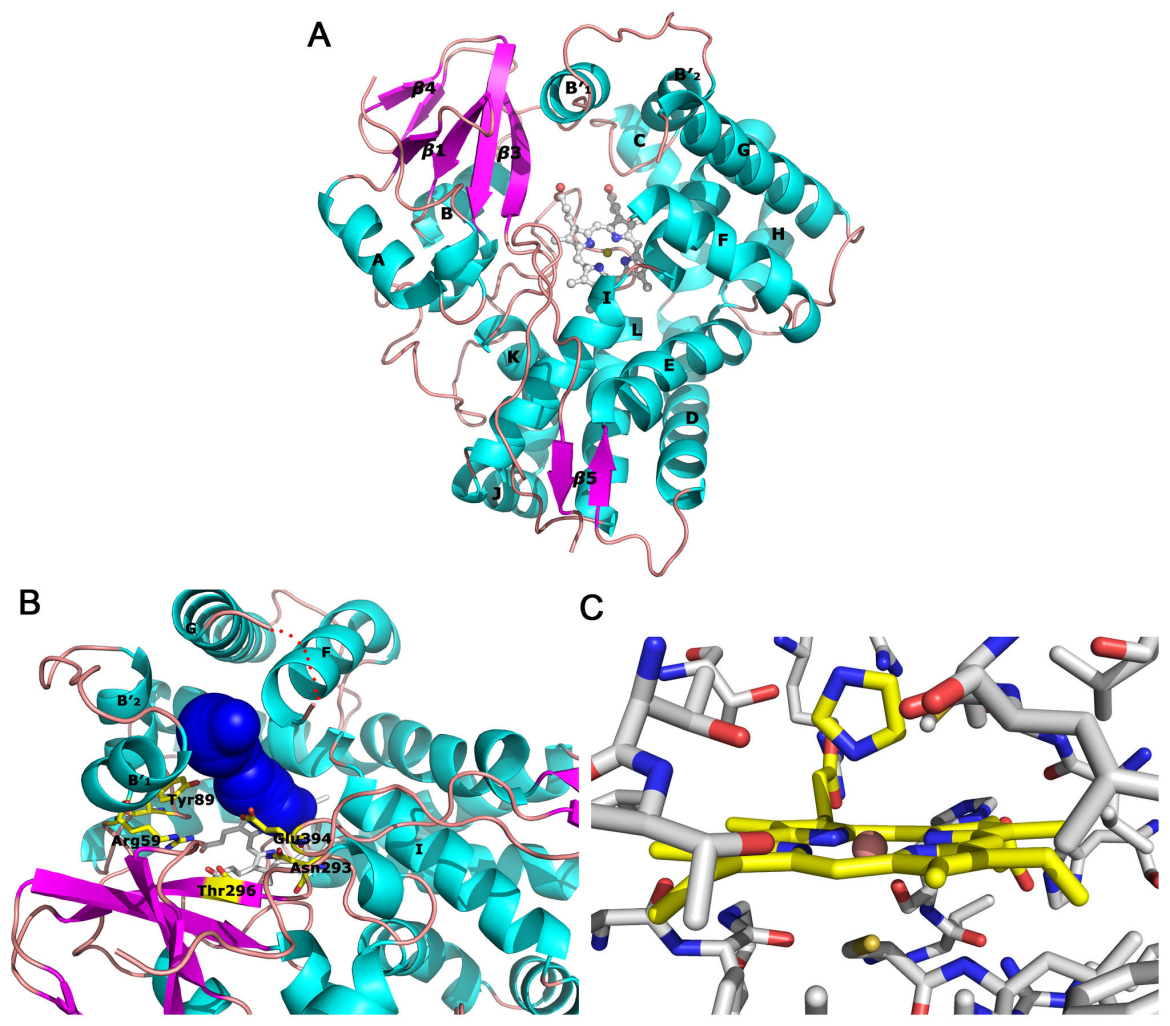
Structure of TxtE. A Overall structure of TxtE. The helices and strands are noted. B The substrate access channel is present as blue tunnel and locates between the B′_1_ helix, the F helix and the G helix, which can be classified as channel 2a according the common nomenclature. Arg59, Tyr89, Asn293, Thr296 and Glu394 are also present. F-G loop is draw as red dots. C Imidazole coordinates to the iron atom of heme.

The purified wild-type TxtE used in this study is catalytically active. L-4-nitrotryptophan could be separated and identified in the reaction products using LC-MS (Figure S1, Supporting Information). The TxtE crystals belong to space group P1, with unit-cell parameters *a* = 46.3 Å, *b* = 52.6 Å, *c* = 88.2 Å, α = 77.8°, β = 82.2° and γ = 68.4°. Detailed crystallographic data statistics for TxtE are shown in Table 1. Although gel filtration results show that TxtE is a monomer in the solution, two molecules are found in the asymmetric unit. TxtE presents a typical trigonal-prism like cytochrome P450 fold with a length of ~50 Å and a thickness of ~30 Å. The overall structure of TxtE is shown in Figure 2A. The N-terminal His-tag, residues 1 of chain A, residues 1 - 4 of chain B, residues 175 - 183 and the last residue of both chains cannot be observed as there is no clear electron density for these residues. This structure also contains three imidazole molecules, two of which are coordinated with the iron atoms of heme. The third imidazole molecule binds with Glu187 of both chains simultaneously.

Compared with the arrangement of the secondary structure elements in the P450 fold, four of the five β-strands (β1, β3, β4 and β5) in TxtE (Figure 2A) can be traced following the order first defined in the structure of CYP101A1[23]. Because of the absence of backbone hydrogen bonds, β2 strand is replaced by a loop in TxtE. Most of α-helices (A - L) sit at positions commonly found in the P450 fold. However, two B′ helices (B′_1_ and B′_2_) are separated by an inserted loop (residues 69 - 77). The helix B′_1_ of TxtE is well overlapped with helix B′ of CYP101A1. The helix B′_2_ locates at the similar position of the B-C loop of CYP101A1, and is parallel to the helix B′_1_ of TxtE. Among the reported CYP structures, it is unusual to find two B′ helices separated by a short loop. Two B′ helices together with B′_2_-C loop, F helix and G helix are located in the distal side of the heme, and are involved in substrate access and binding. In both molecules, the F-G loop (residues 175 - 183) is disordered. These residues may form a lid on the top surface of the substrate access channel (Figure 2B). This channel is defined by Arg59, Val63, Lys67, Phe68 from the B′_1_ helix; Phe79 and Trp82 from the B′_2_ helix; Met88, Tyr89 from the B′_2_C loop; Met173, Thr174 from the F helix; Val184, Thr185, Ile189 from the G helix; Leu241, Ala245 and Thr250 from the I helix; and Asn293, Thr296, Trp297 and Glu394 abound β5 strand. Residues Arg59, Met88, Leu241, Ala245, Thr250, Asn293, Thr296 and Glu394 sit near the bottom of the channel, closely to the heme, and therefore are considered to be a part of the substrate binding pocket. 

### The structure of active site and L-tryptophan binding

The orientation of heme in TxtE is similar to that in other CYPs (Figure 2C). The thiolato-sulfur of Cys357 is the proximal ligand (Fe–S = 2.65 Å). Imidazole is the sixth heme ligand (Fe–N = 2.74 Å). 

**Figure 3:**
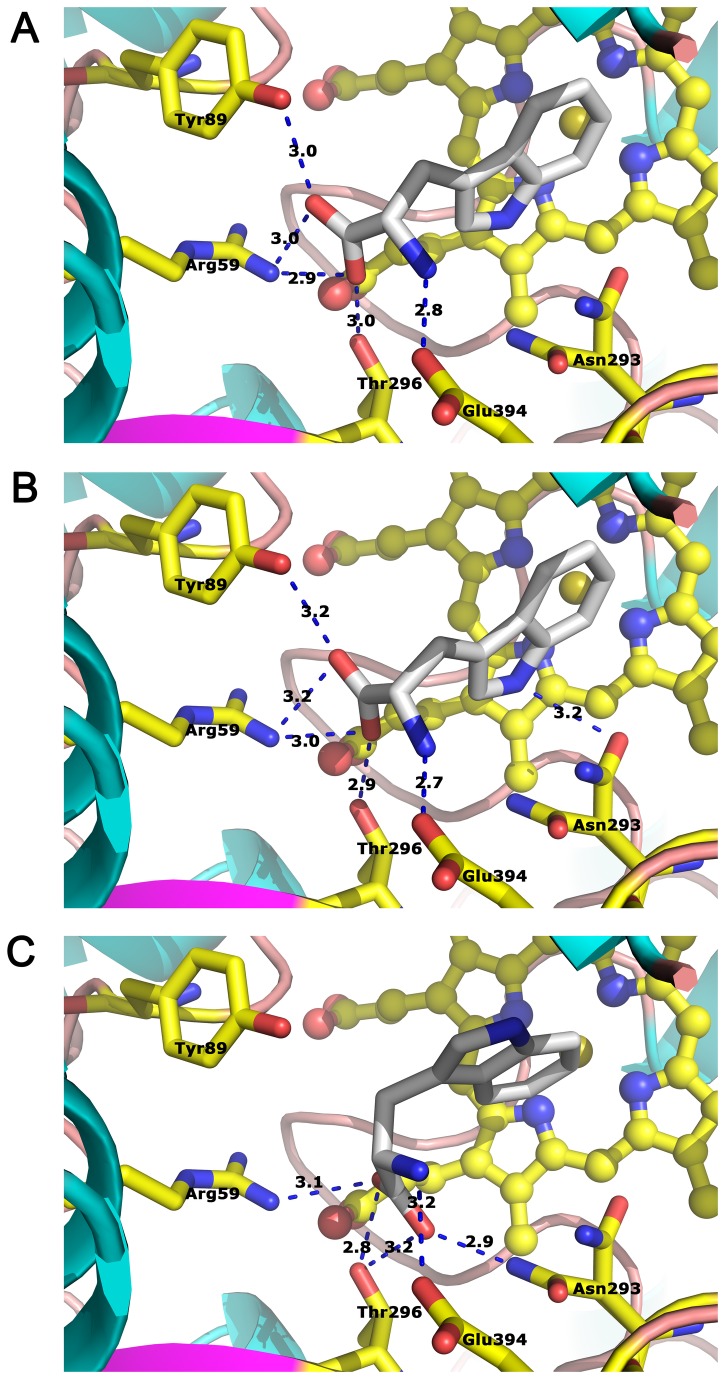
The molecular docking results. A, B and C are the three lowest binding poses, respectively. The polar contacts are presented as blue dashes and distances are also labeled.

Because we could not find crystallization conditions without imidazole, attempts to co-crystallize TxtE with L-tryptophan were unsuccessful. The program AutoDock was therefore used to explore potential L-tryptophan binding poses. Three lowest-energy docking solutions from 2,500,000 searching results were chosen (Figure 3). The calculated binding energies were between –4.63 and –4.95 kcal/mol, and the relative binding poses were similar, especially for the first two poses. Interestingly, we found that, in all three poses, the substrate L-tryptophan carboxylate group interacted with the guanyl group of Arg59 and the hydroxy group of Thr296, and its amino group interacted with the carboxylate group of Glu394 via salt bridges or hydrogen bonds (Figure 3). Besides these three residues, Tyr89 could form polar contacts with the carboxylate group of L-tryptophan, and Asn293 could contact with the imino or carboxylate group of L-tryptophan. 

**Figure 4:**
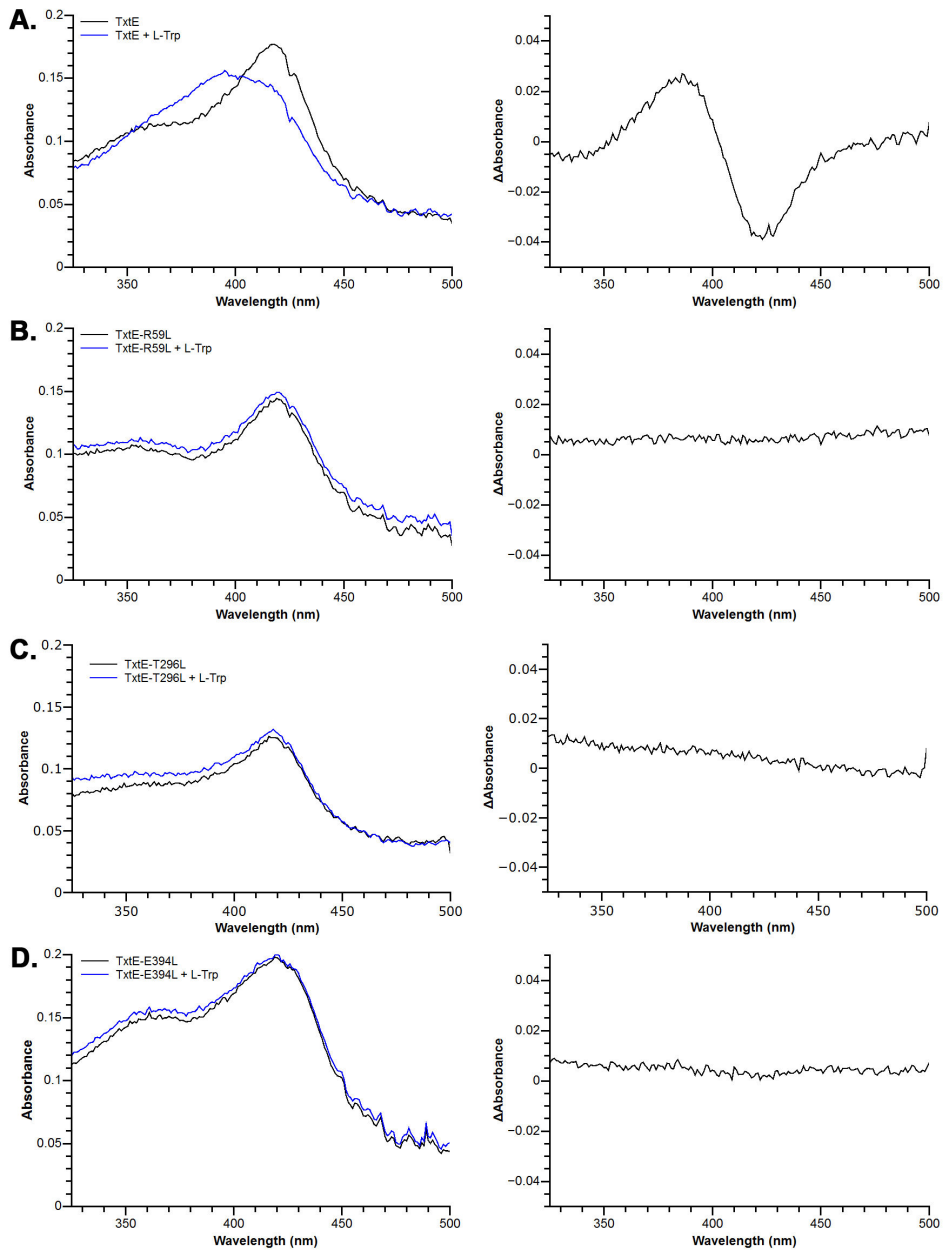
L-tryptophan binding assays of wild-type TxtE, TxtE-T296L and TxtE-E394L. A The UV-vis spectra of TxtE with or without L-tryptophan (Left). TxtE shows an obvious type I different spectrum (Right). B The UV-vis spectra of TxtE-R59L with or without L-tryptophan (Left). No shift can be observed (Right). C The UV-vis spectra of TxtE-T296L with or without L-tryptophan (Left). No shift can be observed (Right).D The UV-vis spectra of TxtE-E394L with or without L-tryptophan (Left). No shift can be observed (Right).

**Figure 5:**
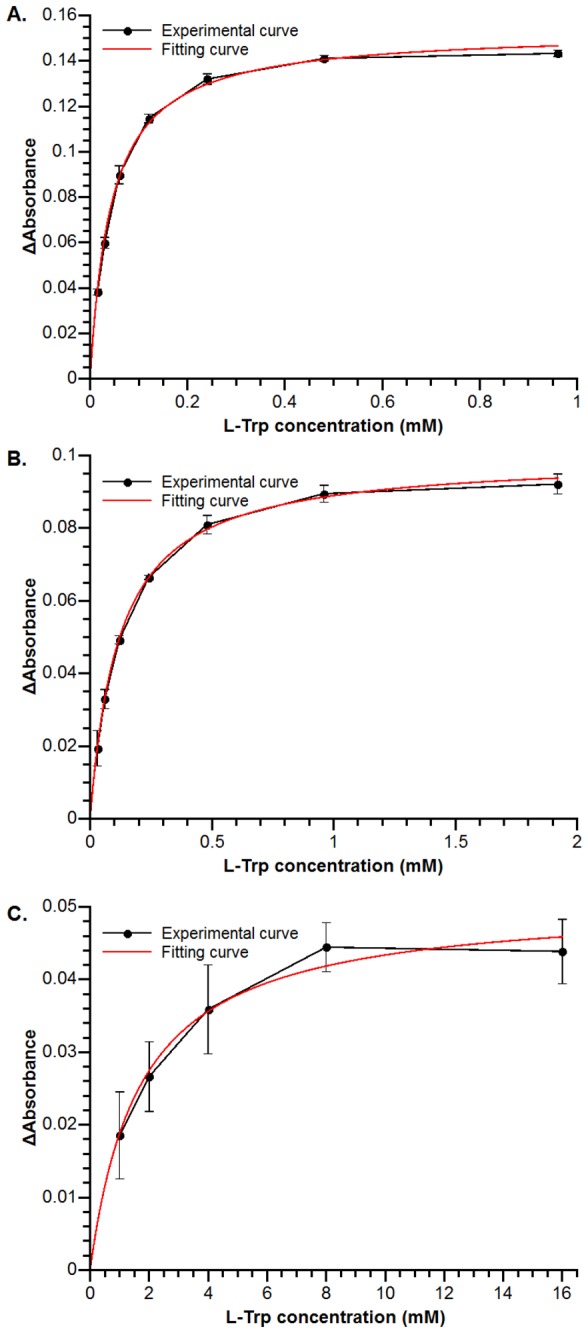
Dissociation constants of L-Tryptophan from wild-type TxtE, TxtE-Y89F and TxtE-N293L. The average values of the difference in absorbance of each spectrum at 390 nm and 420 nm were plotted against the concentration of the substrate. A Wild-type TxtE (8 μM) was titrated with L-tryptophan from 15 μM to 960 μM. B TxtE-Y89F (8 μM) was titrated with L-tryptophan from 30 μM to 1920 μM. C TxtE-N293L (8 μM) was titrated with L-tryptophan from 1 mM to 16 mM.

To validate this docking model of L-tryptophan binding, we designed five single-residue mutation experiments. In which, Arg59, Asn293, Thr296 and Glu394 were mutated to leucine, and Tyr89 was mutated to phenylalanine. In principle, substrate binding of CYPs results in a shift in the UV-vis spectrum of the heme chromophore within the protein[24]. In the type I binding spectrum, the peak is blue-shifted. After substrate binding, the maximum absorption wavelength changes from 420 nm to 390 nm. In this case, the substrate does not coordinate to the iron atom of the heme. In the type II binding spectrum, the peak is red-shifted. Upon substrate binding, the maximum absorption wavelength changes from 420 nm to 420 - 435 nm, which indicates substrate binds directly to the iron atom of the heme. The wild-type TxtE shows a type I binding spectrum after L-tryptophan added. As shown in Figure 4A, after adding L-tryptophan to TxtE solution, the absorption at 390 nm increased, while the absorption at 420 nm decreased. For TxtE-R59L, TxtE-T296L and TxtE-E394L mutants, no shift was observed after L-tryptophan added (Figure 4B, 4C and 4D), which suggests that Arg59, Thr296 and Glu394 are essential for binding the substrate L-tryptophan. TxtE-Y89F and TxtE-N293L still could bind L-tryptophan, but the binding ability decreased significantly, especially for TxtE-N293L. The dissociation constants (K_d_) of TxtE-Y89F and TxtE-N293L were 120.63 ± 4.30 μM and 1.70 ± 0.28 mM, respectively, while the K_d_ of the recombinant wild-type TxtE used in this study was 43.59 ± 2.26 μM (Figure 5). Therefore Asn293 is also likely a key residue for binding L-tryptophan.

### The proton and electron transfer pathway

**Figure 6:**
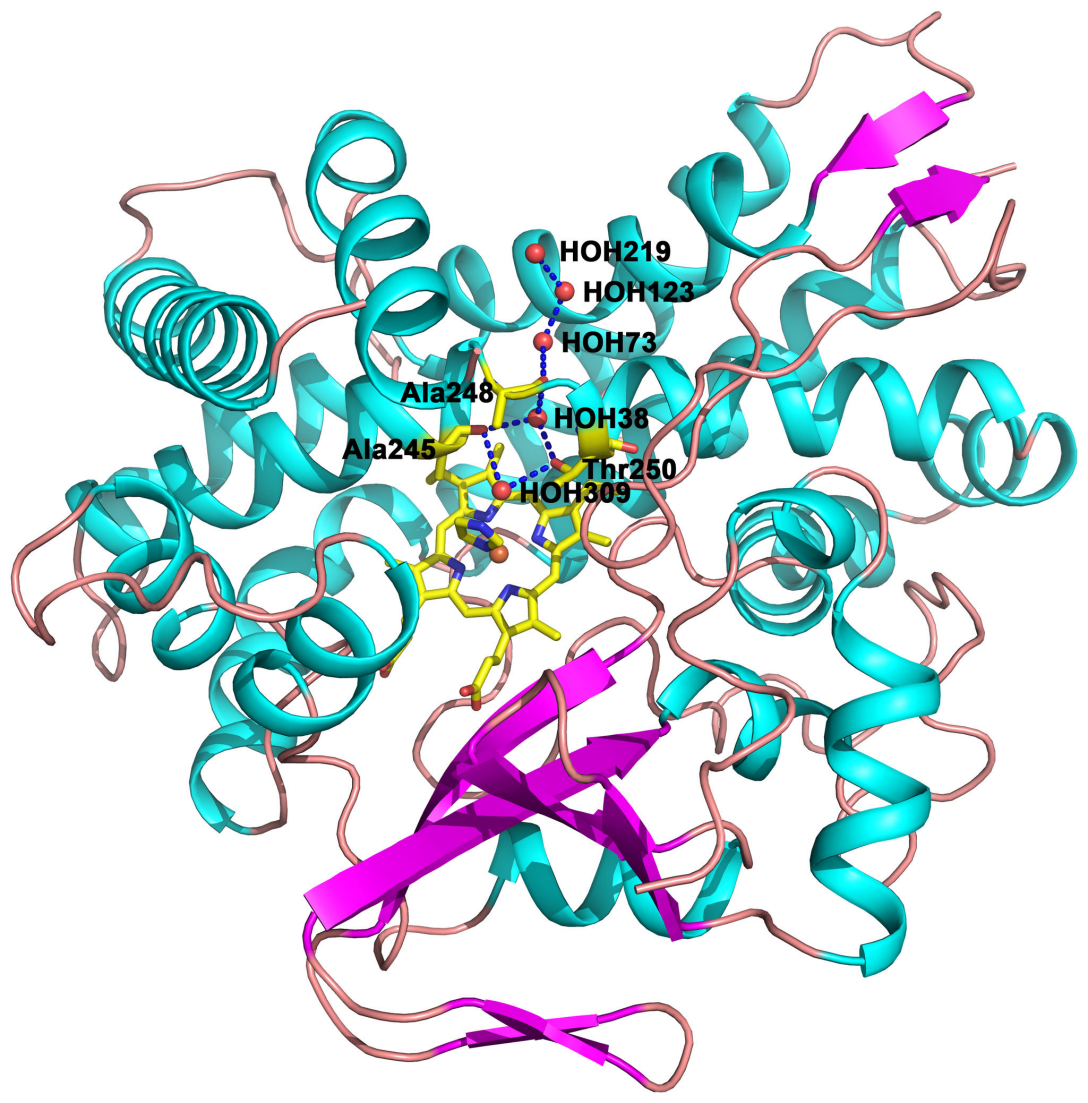
The potential protons transfer pathway. The kink between Ala245 and Thr250 is widened and opens up an entrance between the active site and the external solvent. The hydrogen bonds are presented as blue dashes.

Residues Ala245 - Thr250 in TxtE, which are located at helix I, strongly overlap with Gly248 - Thr252 in CYP101A1. In CYP101A1, the hydrogen bond between the Thr252 hydroxyl group and the Gly248 carbonyl group causes the kink of helix I, which is common in CYPs, and is believed to play important roles in oxygen activation[25]. In contrast to CYP101A1, in TxtE there is one more residue between Ala245 and Thr250. Therefore, the kink between Ala245 and Thr250 is widened which opens up the entrance between the active site and the external solvent (Figure 6). In addition, two water molecules (HOH38 and HOH309) are found to participate in hydrogen bond chain formation between the Thr250 hydroxyl group and the Ala245 carbonyl group. 

Proton transfer is required for the CYPs functions. In the case of TxtE, protons are likely to protonize an Fe(III)-OH intermediate, which is formed at the end of the reaction cycle, to regenerate the resting state of the enzyme. However, in many known CYP structures, the complete proton transfer pathway is not clear with the exception of CYP158A2[26] and P450BM3[27]. Interestingly, in the structure of TxtE a continuous hydrogen bond chain from the active site to the external solvent is observed due to the I helix distortion. This hydrogen bond chain is from HOH309 to HOH219. The carbonyl group of Ala248 forms hydrogen bonds with HOH38 and HOH73 simultaneously. HOH73 links the external solvent (HOH123 and HOH219). HOH309 is near the active site, and links to HOH38 by the carbonyl group of Ala245 and the hydroxyl group of Thr250. However, the hydrogen bond network, as well as the arrangement and number of water molecules in such putative proton transfer pathways, are known to change in the different reaction stages.

The heme proximal face, where the iron atom is closest to the enzyme surface, is the likely binding site for the electron transfer complex. *In vitro*, TxtE can accept electrons from spinach ferredoxin (Fd) and ferredoxin reductase (Fr). Usually, in P450 enzyme reactions, there are two electrons transferred from NAD(P)H through the Fd and FrFr pathway, which can be divided into two transfer processes. The first one happens between the CYP-substrate complex and Fd, and the second one happens between CYP-dioxygen-substrate complex and Fd. According to the theoretical and experimental results, in CYP101A1, Arg112 plays important roles in the first electron transfer[28]. Compared with the heme proximal face residues of TxtE and CYP101A1, we found that Arg112 is located at the identical location in TxtE. This implies that TxtE may have the same first electron transfer pathway as that in CYP101A1. 

## Discussion

Although the overall folds of CYPs are strongly conserved, the precise position of various structure elements differs substantially, especially in the regions controlling substrate specificity. In the structure reported here, TxtE has the characteristic P450 fold. A clearly defined substrate access channel was observed between helix B′_1_ and helix G which can be classified as channel 2a according the common nomenclature[29]. Channel 2a is common in bacteria CYPs. TxtE has two B′ helices, which is an uncommon conformation. Besides TxtE, there are five known CYP structures which have two B′ helices, but only AurH[30], CYP121[31] and CalO_2_[32] form two B′ helices bundle similar to TxtE. This B′ helices bundle creates a hydrophobic “wall” that shields the substrate binding pocket from solvent, and seals the substrate binding pocket. Other examples with two B′ helices are CYP119[33] and P450epoK[34]. In CYP119 B′_2_ helix only has one turn, and in P450epoK these two helices are nearly vertical. 

In the reaction of CYPs, protons should reach the active site through a hydrogen bond chain involving water molecules. Unfortunately, few CYP structures show a clear proton transfer pathway from the active site to the external solvent except CYP158A2[26] and P450BM3[27]. The continuous hydrogen bond chains from the active site to the external solvent were observed in the CYP158A2-dioxygen-substrate complex and the P450BM3-reductase domain complex, in which the hydrogen bond chain is nearly vertical to the heme plane. Compared with these two structures, TxtE shows a unique proton transfer pathway which crosses the helix I distortion and is nearly parallel to the heme plane. The mechanism of oxygen activation of CYP101A1 was elaborated [25]. TxtE shows a possible mechanism of oxygen activation, which is consistent with that of CYP101A1. In CYP101A1, Thr252_CYP101A1_ and a water molecule are required to form hydrogen bonds with dioxygen simultaneously for the cleavage of dioxygen. In TxtE, Thr250 and HOH309 may have the same roles in oxygen activation although the precise positions of HOH309 and Thr250 may be changed after dioxygen binding due to the rearrangement of water molecules. In CYP101A1, protons are also transferred through widened helix I, but the water chain only extend to Glu366_CYP101A1_. Compared with CYP101A1, the kink of the helix in TxtE is wider than that in CYP101A1. Therefore it is easier to form a complete water chain through this kink in the reaction process.

The molecular docking suggests that carboxyl group and amino group of L-tryptophan form salt bridges or hydrogen bonds to Arg59, Tyr89, Asn293, Thr296 and Glu394. The bond lengths (from proton donor to accepter) are shown in Figure 3. Substrate binding assays show Arg59, Asn293, Thr296 and Glu394 are key residues for binding L-tryptophan. In our simulation results, the distance between the carbon atom at 4-position of indolyl moiety and the iron atom of heme is between 5.50 and 6.22 Å, which is a reasonable distance in CYPs. To date, it has not been possible to obtain crystals of the substrate-bound form of TxtE, so we cannot determine whether this conformation of the side chain is the actual conformation in protein-substrate complex. Even so, because tryptophan has the largest side chain in all of amino acids, no side chain atom can be at the corresponding position of 4-position of L-tryptophan, even with the conformation of the side chains changing. In this proposed model, the formation of the TxtE-substrate complex mainly depends on the amino and carboxyl groups of substrates, but not the side chain. This provides the possibility to modify TxtE or substrates for producing nitration products of amino acids analogs. Nitration products of amino acids and analogs are important drug synthesis intermediates. Our findings can help to develop enzymatic synthesis pathway of these intermediates, and avoid the risk of chemical synthesis. However, our docking model is based on the substrate-free conformation. CYPs usually undergo a conformational change upon substrate binding. To clarify the substitute binding mechanism of TxtE completely, it still need to determine the crystal structure of TxtE and L-tryptophan complex.

## Supporting Information

Figure S1 **The enzyme activity assay.** The enzyme reaction products were analyzed by using LC-MS. The *m/z* of L-tryptophan is 205, and *m/z* of L-4-nitrotryptophan is 250. Ions at *m*/*z* = 250 were detected in the products which were catalyzed by using un-boiled TxtE, but could not be detected in the products which were catalyzed by using boiled TxtE.
(PDF)

Materials and Methods S1 (PDF)
