# Supplementary material for: Structural Insights into the Mechanism for Recognizing Substrate of the Cytochrome P450 Enzyme TxtE
Source: PLoS One. 2013 Nov 25;8(11):e81526. doi: 10.1371/journal.pone.0081526 (PMC3840065; doi:10.1371/journal.pone.0081526)
Supplement: Figure S1 — The enzyme activity assay. The enzyme reaction products were analyzed by using LC-MS. The m/z of L-tryptophan is 205, and m/z of L-4-nitrotryptophan is 250. Ions at m/z = 250 were detected in the products which were catalyzed by using un-boiled TxtE, but could not be detected in the products which were catalyzed by using boiled TxtE. (PDF) [file pone.0081526.s001.pdf]

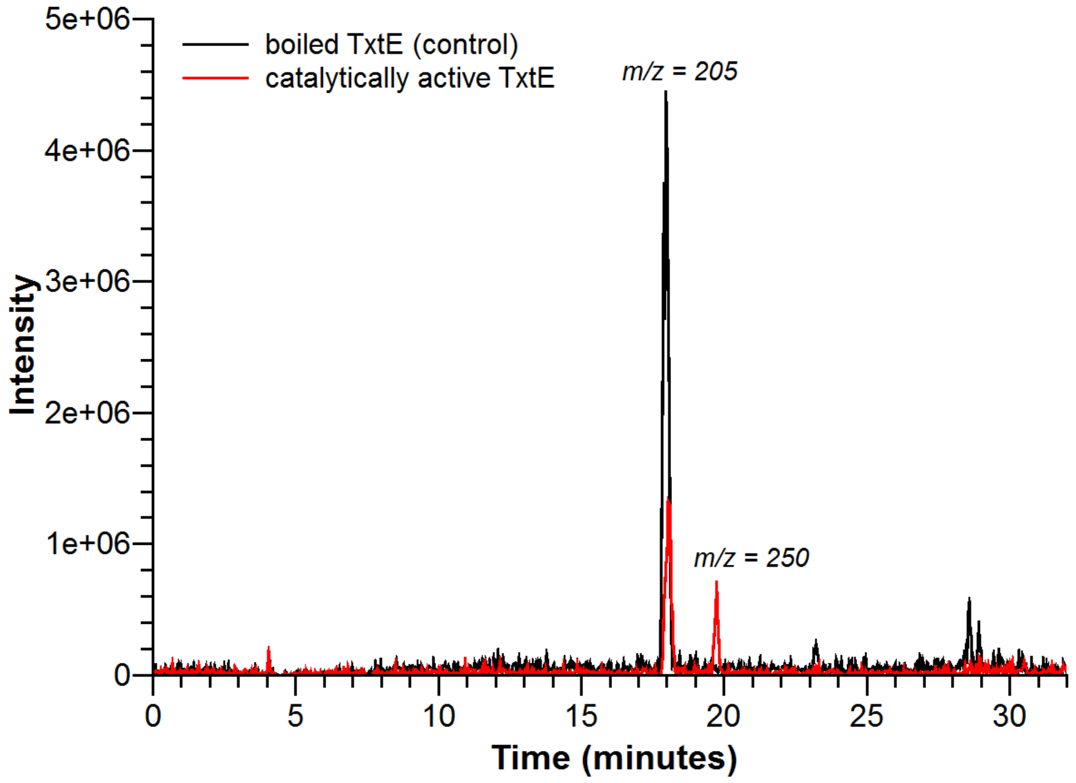

Figure S1. The enzyme activity assay. The enzyme reaction products were analyzed by using LC-MS. The *m/z* of L-tryptophan is 205, and *m/z* of L-4-nitrotryptophan is 250. Ions at *m/z* = 250 were detected in the products which were catalyzed by using un-boiled TxtE, but could not be detected in the products which were catalyzed by using boiled TxtE.
