# Supplementary material for: Structural Insights into the Mechanism for Recognizing Substrate of the Cytochrome P450 Enzyme TxtE
Source: PLoS One. 2013 Nov 25;8(11):e81526. doi: 10.1371/journal.pone.0081526 (PMC3840065; doi:10.1371/journal.pone.0081526)
Supplement: Materials and Methods S1 — (PDF) [file pone.0081526.s002.pdf]

Supplementary material

Materials and methods section for the enzyme activity assay shown in Figure S1

The enzyme activity assay was performed as in reference[1]. 1.5 μM TxtE, 0.5 mM DEANO (Diethylamine NONOate sodium salt hydrate), 1 mM NADPH, 2 μg ferredoxin, 0.034 units ferredoxin reductase and 0.5 mM L-tryptophan were incubated in a total volume of 200 μL of Tris buffer (25 mM, pH 8) at room temperature for 120 minutes. 1.3 μL HCl (11.9 M) was added to terminate the reaction and the solution was filtered using 0.22 μm centrifugal filter tubes (Millipore). In control sample, the boiled TxtE was used. After the reaction, solution became yellow, and was analyzed by using LC-MS (Waters 2695 HPLC and Waters ZQ2000 mass detector, Waters, US). The sample was separated by using Waters xBridge C18 column (dimension: 4.6 × 250 mm, particle size: 5 μm). The result was shown in the supplementary figure 1.

1. Barry SM, Kers JA, Johnson EG, Song LJ, Aston PR, et al. (2012) Cytochrome P450-catalyzed L-tryptophan nitration in thaxtomin phytotoxin biosynthesis. Nature Chemical Biology 8: 814-816.
